# Age- and sex-dependent changes of resting amygdalar activity in individuals free of clinical cardiovascular disease

**DOI:** 10.1007/s12350-020-02504-7

**Published:** 2021-01-13

**Authors:** Achi Haider, Susan Bengs, Flavia Diggelmann, Gioia Epprecht, Dominik Etter, Anna Luisa Beeler, Winandus J. Wijnen, Valerie Treyer, Angela Portmann, Geoffrey I. Warnock, Muriel Grämer, Atanas Todorov, Tobias A. Fuchs, Aju P. Pazhenkottil, Ronny R. Buechel, Felix C. Tanner, Philipp A. Kaufmann, Catherine Gebhard, Michael Fiechter

**Affiliations:** 1 Department of Nuclear Medicine, University Hospital Zurich, Raemistrasse 100, 8091 Zurich, Switzerland; 2 Center for Molecular Cardiology, University of Zurich, 8952 Schlieren, Switzerland; 3 Department of Cardiology, University of Zurich, 8091 Zurich, Switzerland; 4 Swiss Paraplegic Center, 6207 Nottwil, Switzerland

**Keywords:** Amygdala, aging, sex differences, heart-brain axis, emotional stress, ^18^F-fluorodeoxyglucose (^18^F-FDG), positron emission tomography (PET), echocardiography, cardiovascular disease, risk stratification

## Abstract

**Purpose:**

Amygdalar metabolic activity was shown to independently predict cardiovascular outcomes. However, little is known about age- and sex-dependent variability in neuronal stress responses among individuals free of cardiac disease. This study sought to assess age- and sex-specific differences of resting amygdalar metabolic activity in the absence of clinical cardiovascular disease.

**Methods:**

Amygdalar metabolic activity was assessed in 563 patients who underwent multimodality imaging by ^18^F-fluorodeoxyglucose (^18^F-FDG) positron emission tomography/computed tomography and echocardiography for the evaluation of cardiac function.

**Results:**

After exclusion of 294 patients with structural or functional cardiovascular pathologies, 269 patients (128 women) remained in the final population. ^18^F-FDG amygdalar activity significantly decreased with age in men (*r* = − 0.278, *P *= 0.001), but not in women (*r* = 0.002, *P *= 0.983). Similarly, dichotomous analysis confirmed a lower amygdalar activity in men ≥ 50 years as compared to those < 50 years of age (0.79 ± 0.1 vs. 0.84 ± 0.1, *P *= 0.007), which was not observed in women (0.81 ± 0.1 vs. 0.82 ± 0.1, *P *= 0.549). Accordingly, a fully adjusted linear regression analysis identified age as an independent predictor of amygdalar activity only in men (B-coefficient − 0.278, *P *= 0.001).

**Conclusion:**

Amygdalar activity decreases with age in men, but not in women. The use of amygdalar activity for cardiovascular risk stratification merits consideration of inherent age- and sex-dependent variability.

**Supplementary Information:**

The online version of this article (10.1007/s12350-020-02504-7) contains supplementary material, which is available to authorized users.

## Introduction

Cardiovascular disease remains the primary cause of death in Europe, accounting for mortality rates of 49% and 40% in women and men, respectively.^1^ In particular, there is increasing evidence for sex differences in the pathophysiology of coronary artery disease (CAD), including the female propensity towards non-obstructive CAD, coronary microvascular dysfunction, and stress (Takotsubo) cardiomyopathy, thus prompting reassessment of traditional cardiovascular risk conditions.^2^ As such, considerable efforts have been devoted to understand how emotional stress is translated into adverse cardiovascular outcomes.

The brain’s salience network, an ensemble of interconnected brain regions responsible for emotional stress responses, has recently gained increasing attention within the context of CAD. Indeed, a recent landmark study demonstrated that the resting amygdalar metabolic activity, a key element of the brain’s salience network, independently predicted cardiovascular outcomes.^3^ Further, increased amygdalar activity was linked to impaired myocardial perfusion and function in women, but not in men, which is in agreement with the higher incidence of stress cardiomyopathy in women.^4^,^5^ Despite the detrimental association of enhanced amygdalar activity in CAD patients, little is known about neuronal stress responses in individuals free of cardiac disease. Understanding how the resting amygdalar activity is affected by age and sex in the absence of cardiac disease, however, is crucial for accurate risk assessment in CAD patients. Thus, we sought to assess age- and sex-specific differences of amygdalar metabolic activity in individuals free of clinical cardiovascular disease.

## Methods and Results

A total of 563 patients underwent clinically indicated oncologic whole-body ^18^F-fluorodeoxyglucose (^18^F-FDG) positron emission tomography (PET)/computed tomography (CT) and echocardiography to assess cardiac function according to current guidelines.^6^ After exclusion of 294 patients with impaired left ventricular ejection fraction (LVEF < 50%), presence of wall-motion abnormalities, history of coronary artery disease (PCI, CABG, previous myocardial infarction), and/or diabetes, 269 patients (128 [48%] women) free of clinical cardiovascular disease remained in the final study population. None of these patients had metabolically active malignancies. After measurement of patient’s blood glucose level (5.5 ± 1.2 mmol/L in women and 5.8 ± 1.4 mmol/L in men, *P* = 0.11 for women vs. men), a dose of ~ 350 MBq ^18^F-FDG was injected into a peripheral vein. Following tracer injection, individuals were allowed to rest for 45-60 minutes before a standardized PET image acquisition protocol, which includes non-contrast CT, was applied. Whole-body scans were cropped for the skull and quantitative assessment of resting amygdalar activity was performed as previously described.^4^ Briefly, serial regions of interest (ROI) were placed in both amygdalae to measure ^18^F-FDG standardized uptake value (SUV) by use of PMOD software V4.1 (PMOD Technologies LLC, Zurich, Switzerland) and application of the brain maximum probability map (Hammersmith atlas,^7^). Normalization was performed against cerebellar activity. PET/CT and echocardiography were performed within a maximum time frame of 3 months. Pearson’s *r* was applied to investigate associations, whereas linear regression models were used to identify predictor variables. The study was reviewed and approved by the institutional review board (BASEC No. 2017-01112). The ethics committee waived the need for informed consent due to the retrospective study design.

**Figure 1:**
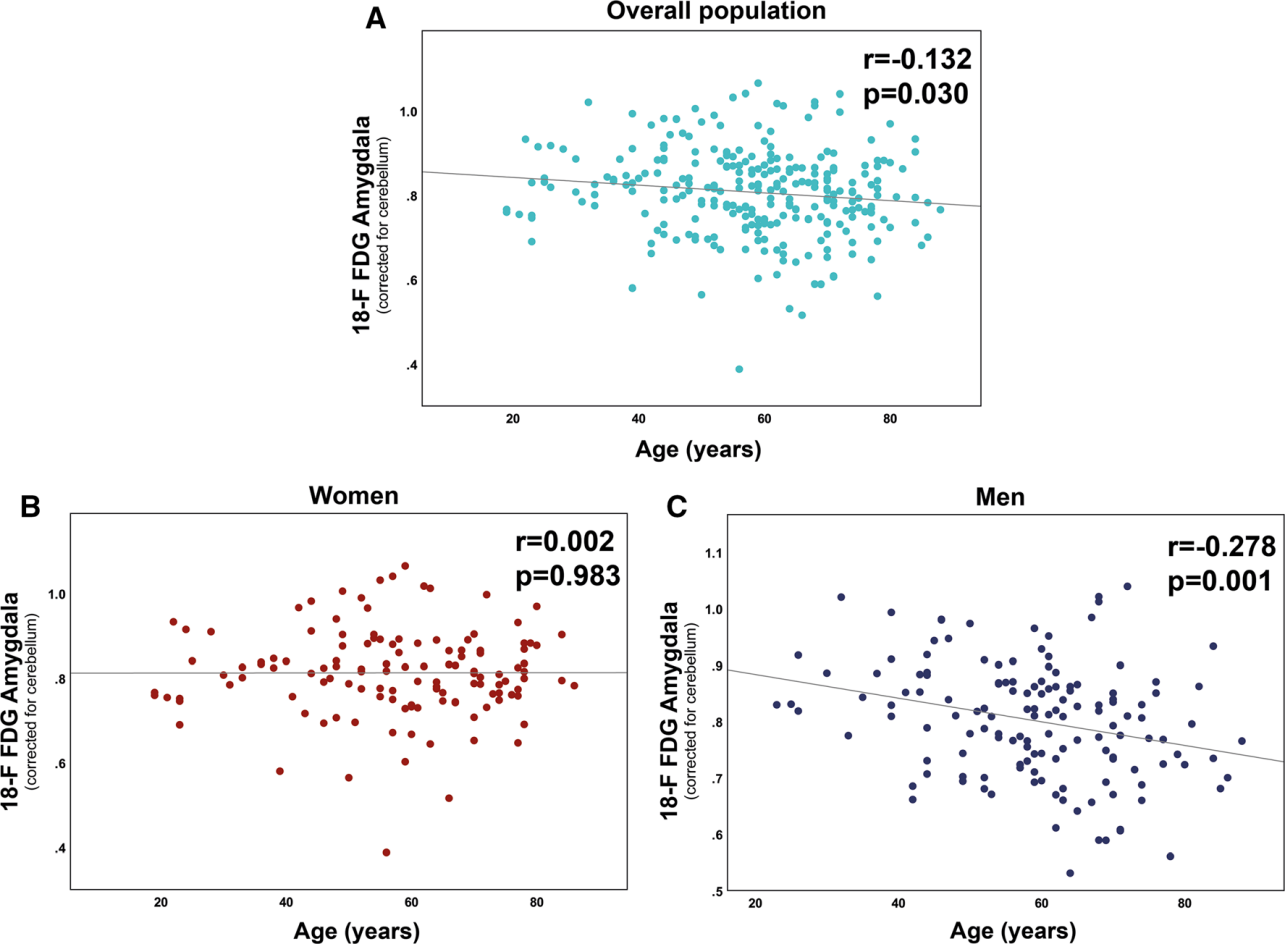
Correlation of amygdalar activity with age. **A** Pearson product-moment correlation reveals a gradual decline of amygdala metabolism during the aging process (*P* = 0.030) in the whole study population. **C** When stratified by sex, this correlation was significant in men (*P* = 0.001). **B** No correlation of amygdalar activity with age was observed in women (*P* = 0.983)

**Figure 2:**
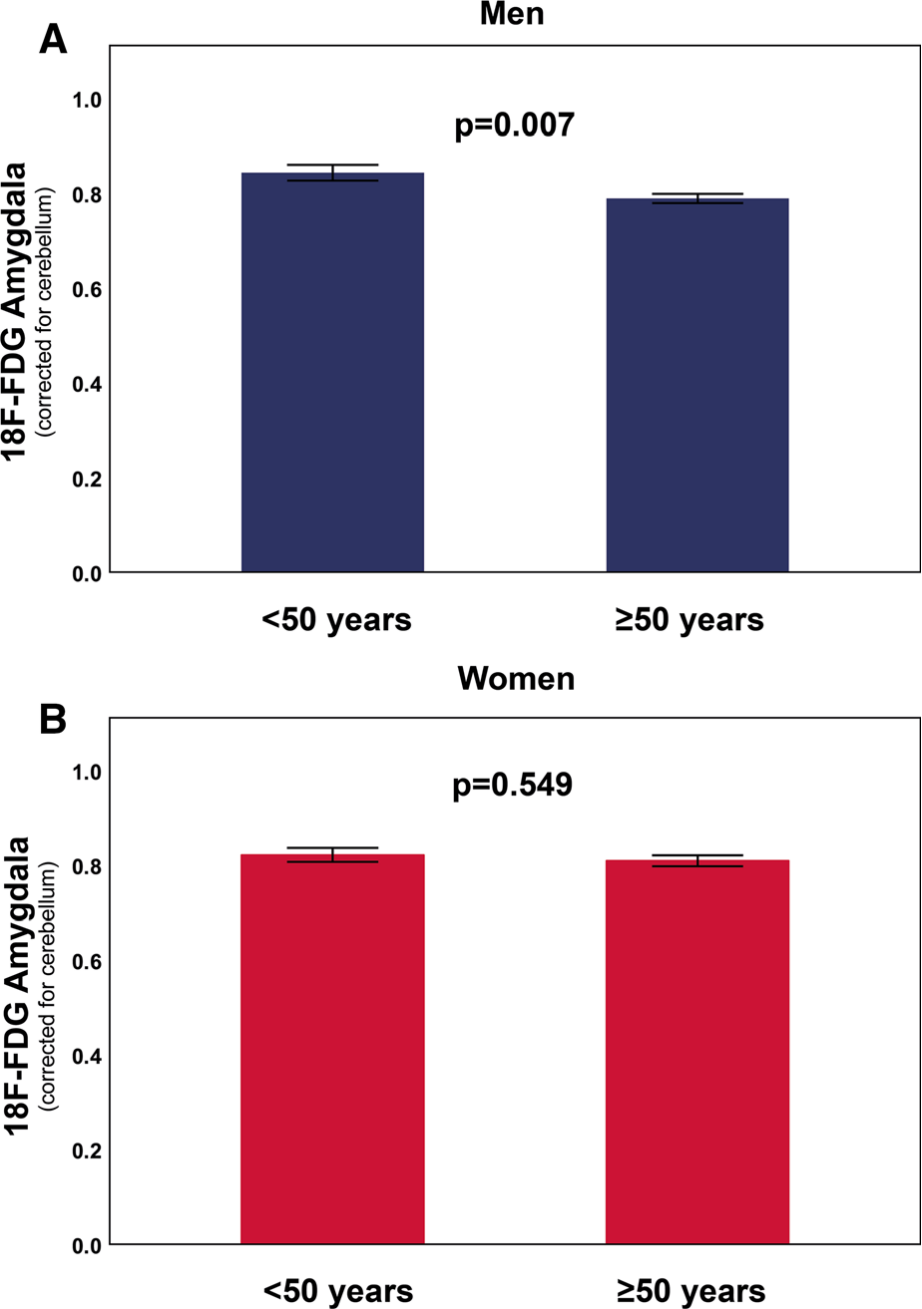
Age-dependent dichotomous assessment of resting amygdalar activity in men and women. **A** Amygdalar activity is significantly lower in men ≥ 50 years of age (*P* = 0.007). **B** No age-dependent changes in amygdalar activity are observed in women (*P* = 0.549). Error bars indicate standard error, SE. *P*-values are depicted

As depicted in Table 1, patient baseline characteristics were comparable between women and men, with the exceptions of smoking (women vs. men, 9.4% vs. 20.6%, *P *= 0.011), left ventricular ejection fraction (LVEF, women vs. men, 62.9 ± 5.5 vs. 60.7 ± 6.0, *P *= 0.002), and creatinine levels (women vs. men, 85.1 ± 79.2 vs. 118.4 ± 97.5, *P *= 0.028). The mean age was 57.4 ± 16.3 years in women and 59.0 ± 13.5 years in men. Pearson’s *r* correlation analysis showed a weak and negative association between age and ^18^F-FDG amygdalar uptake in the whole study population (*r *= − 0.132, *P *= 0.030, Figure 1A). When stratified by sex, this relation was not observed in women (*r* = 0.002, *P *= 0.983, Figure 1B), but was pronounced and inverse in men (*r* = − 0.278, *P *= 0.001, Figure 1C). Dichotomous analysis confirmed a significantly lower amygdalar activity in men ≥ 50 years as compared to those < 50 years of age (0.79 ± 0.1 vs. 0.84 ± 0.1, *P *= 0.007, Figure 2A). In contrast, there was no difference in amygdalar activity in women ≥ 50 vs. < 50 years of age (0.81 ± 0.1 vs. 0.82 ± 0.1, *P *= 0.549, Figure 2B). Stepwise linear regression analysis for ^18^F-FDG amygdalar uptake as a dependent variable and age, hypertension, dyslipidemia, obesity, positive family history for CAD, or smoking as predictor variables identified age as sole independent predictor for amygdalar activity in men (*B*-coefficient − 0.278, *P *= 0.001). In the whole study population, an interaction term consisting of age and male sex was selected as an independent predictor for amygdalar activity in a linear regression model (*B*-coefficient − 0.200, *P *= 0.001).

**Table 1:** Patient baseline characteristics

Patient baseline characteristics	Total n = 269	Women n = 128	Men n = 141	*P*-value (women vs. men)
Age (years), mean±SD	58.2 ± 14.9	57.4 ± 16.3	59.0 ± 13.5	0.397
Hypertension, n(%)	82 (30.5)	39 (30.5)	43 (30.5)	1.000
Dyslipidemia, n(%)	18 (6.7)	7 (2.6)	11 (4.1)	0.475
Obesity, n(%)	39 (14.5)	19 (14.8)	20 (14.2)	1.000
Family history of CAD, n(%)	3 (1.1)	1 (0.8)	2 (1.4)	1.000
Smoking, n(%)	41 (15.2)	12 (9.4)	29 (20.6)	0.011
Alcohol, n(%)	18 (6.7)	6 (4.7)	12 (8.5)	0.232
LVEF (%), mean±SD	61.2 ± 5.8	62.9 ± 5.5	60.7 ± 6.0	0.002
Blood glucose (mmol/l), mean±SD	5.6 ± 1.3	5.5 ± 1.2	5.8 ± 1.4	0.105
Creatinine (µmol/l), mean±SD	102.3 ± 90.4	85.1 ± 79.2	118.4 ± 97.5	0.028
CRP (mg/l), mean±SD	53.5 ± 68.8	47.0 ± 66.6	59.2 ± 70.8	0.302
NT-proBNP (ng/l), mean±SD	1553 ± 2468	1204 ± 2524	2018 ± 2418	0.398
Neutrophils (x10^3^/µl), mean±SD	5.8 ± 3.5	6.2 ± 4.1	5.5 ± 2.9	0.237
Lymphocytes (x10^3^/µl), mean±SD	1.3 ± 0.8	1.4 ± 0.8	1.3 ± 0.7	0.159
^18^F-FDG bone marrow (SUV), mean±SD	1.9 ± 0.5	1.9 ± 0.5	1.9 ± 0.5	0.434
^18^F-FDG amygdala (rSUV), mean±SD	0.8 ± 0.1	0.8 ± 0.1	0.8 ± 0.1	0.388
ACE, n(%)	38 (14.2)	20 (15.6)	18 (12.9)	0.600
ARBs, n(%)	38 (14.2)	17 (13.3)	21 (15.1)	0.728
Beta blockers, n(%)	65 (24.3)	30 (23.4)	35 (25.2)	0.777
Loop diuretics, n(%)	41 (15.4)	18 (14.1)	23 (16.5)	0.613
ASS, n(%)	52 (19.5)	23 (18.0)	29 (20.9)	0.643
Antidepressants, n(%)	31 (11.6)	16 (12.5)	15 (10.8)	0.705
Statin, n(%)	42 (15.7)	21 (16.4)	21 (15.1)	0.867
Corticosteroids	66 (24.7)	35 (27.3)	31 (22.3)	0.395

*ACE/ARBs*, angiotensin converting enzyme/angiotensin II receptor blockers; *ASS*, acetylsalicylic acid; *CAD*, coronary artery disease; *CRP*, C-reactive protein; ^*18*^*F-FDG*, ^18^F-fluorodeoxyglucose; *LVEF*, left ventricular ejection fraction; *NT-proBNP*, N-terminal pro-B-type natriuretic peptide; *SD*, standard deviation; *SUV*, standardized uptake value; *rSUV*, relative standardized uptake value. Data are presented as mean ± SD or frequencies (percentage). Two-sided *P*-values are indicated

## Discussion

Taken together, we observed age- and sex-dependent alterations of amygdalar activity in individuals free of clinical cardiovascular disease. While amygdalar activity significantly decreased with age in men, women showed no age-related changes in amygdalar activity. Accordingly, age was selected as an independent negative predictor of amygdalar activity in men, but not in women. These findings imply that the use of resting amygdalar metabolic activity for cardiovascular risk stratification, as suggested in a recent landmark study by Tawakol et al,^3^ merits careful consideration of age and sex as potential confounders.

Chronic psychosocial stress is an established cardiovascular risk factor. Nonetheless, the mechanisms that translate emotional stress into cardiovascular events remain poorly understood.^8^ Among the suggested mechanisms, the concept that increased amygdalar activity results in enhanced hematopoiesis in the bone marrow, thus contributing to arterial inflammation and subsequent worsening of CAD is particularly intriguing.^3^,^9^ Our study implies that amygdalar metabolic activity is attenuated in elderly men, but not in elderly women, suggesting that women exhibit a persistently high neuronal stress level at older age. It is therefore tempting to hypothesize that chronic activation of the brain’s salience network might predispose women to a higher cardiovascular vulnerability in situations of increased demand such as an acute coronary syndrome (ACS). Indeed, recent data indicate that women perceive greater emotional stress following an ACS and are more likely to develop mental-stress induced ischemia than men.^10^ Both observations emphasize the urgent need to account for psychosocial factors in primary and secondary prevention in women.^2^,^11^,^12^

As with any study, certain inherent design limitations should be pointed out. First, this study is a single-center retrospective analysis conducted in a cohort of aged individuals, which limits its generalizability. Second, our study is purely observational, and causality cannot be inferred from the associations shown. Third, although a comprehensive group of adjustment variables was employed, unmeasured factors affecting amygdalar metabolic activity, such as subclinical CAD and other non-detected cardiovascular conditions, as well as baseline differences between men and women, may have affected our endpoints. Fourth, given that patients were not specifically referred for assessment of cardio-neurological associations, a potential selection bias cannot be completely ruled out. Accordingly, the findings of this study are to be considered as hypothesis-generating and need to be confirmed in larger and, ideally, prospective studies.

In conclusion, our findings highlight that naturally occurring age- and sex-dependent changes need to be taken into consideration when using resting amygdalar activity as a risk stratification tool in patients with suspected or known CAD. Whether there is a direct link between adverse cardiovascular outcomes and chronic activation of the brain’s salience network in aging women will have to be assessed in future studies.

## New Knowledge Gained

Amygdalar metabolic activity declines with age in men, but not in women. Naturally occurring age- and sex-dependent changes in amygdalar metabolic activity need to be taken into consideration when using this metric as a risk stratification tool for patients with suspected or known CAD.

## Electronic supplementary material

Below is the link to the electronic supplementary material.

Electronic supplementary material 1 (PPTX 440 kb)

Electronic supplementary material 2 (M4A 6161 kb)
