# Supplementary material for: Age- and sex-dependent changes of resting amygdalar activity in individuals free of clinical cardiovascular disease
Source: J Nucl Cardiol. 2021 Jan 13;28(2):427–32. doi: 10.1007/s12350-020-02504-7 (PMC8076110; doi:10.1007/s12350-020-02504-7)
Supplement: Supplementary file 1 — Electronic supplementary material 1 (PPTX 440 kb) [file 12350_2020_2504_MOESM1_ESM.pptx]

## Slide 1
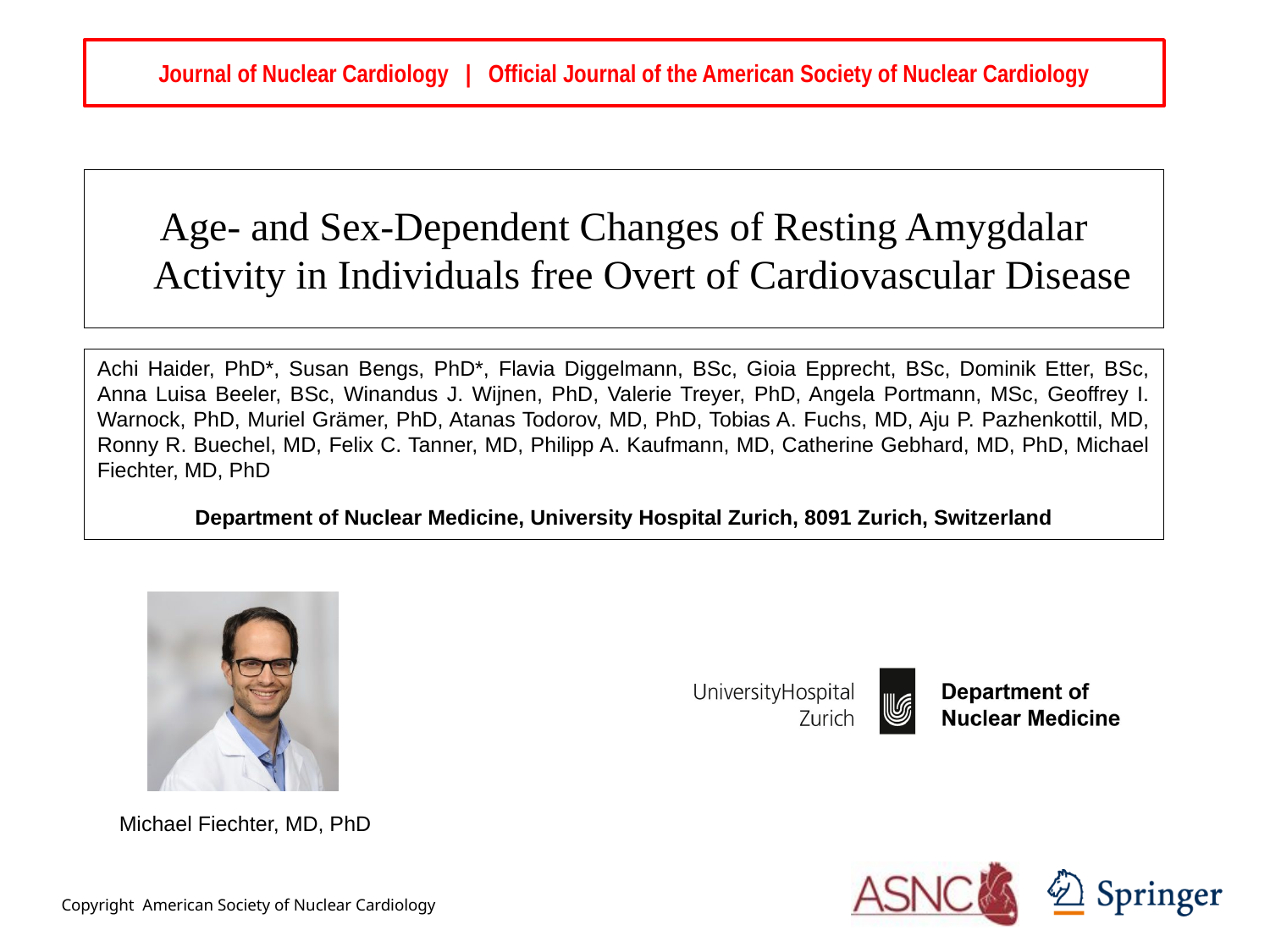

Journal of Nuclear Cardiology | Official Journal of the American Society of Nuclear Cardiology
# Age- and Sex-Dependent Changes of Resting Amygdalar Activity in Individuals free Overt of Cardiovascular Disease
Achi Haider, PhD*, Susan Bengs, PhD*, Flavia Diggelmann, BSc, Gioia Epprecht, BSc, Dominik Etter, BSc, Anna Luisa Beeler, BSc, Winandus J. Wijnen, PhD, Valerie Treyer, PhD, Angela Portmann, MSc, Geoffrey I. Warnock, PhD, Muriel Grämer, PhD, Atanas Todorov, MD, PhD, Tobias A. Fuchs, MD, Aju P. Pazhenkottil, MD, Ronny R. Buechel, MD, Felix C. Tanner, MD, Philipp A. Kaufmann, MD, Catherine Gebhard, MD, PhD, Michael Fiechter, MD, PhD
Department of Nuclear Medicine, University Hospital Zurich, 8091 Zurich, Switzerland
Michael Fiechter, MD, PhD
Copyright American Society of Nuclear Cardiology

## Slide 2
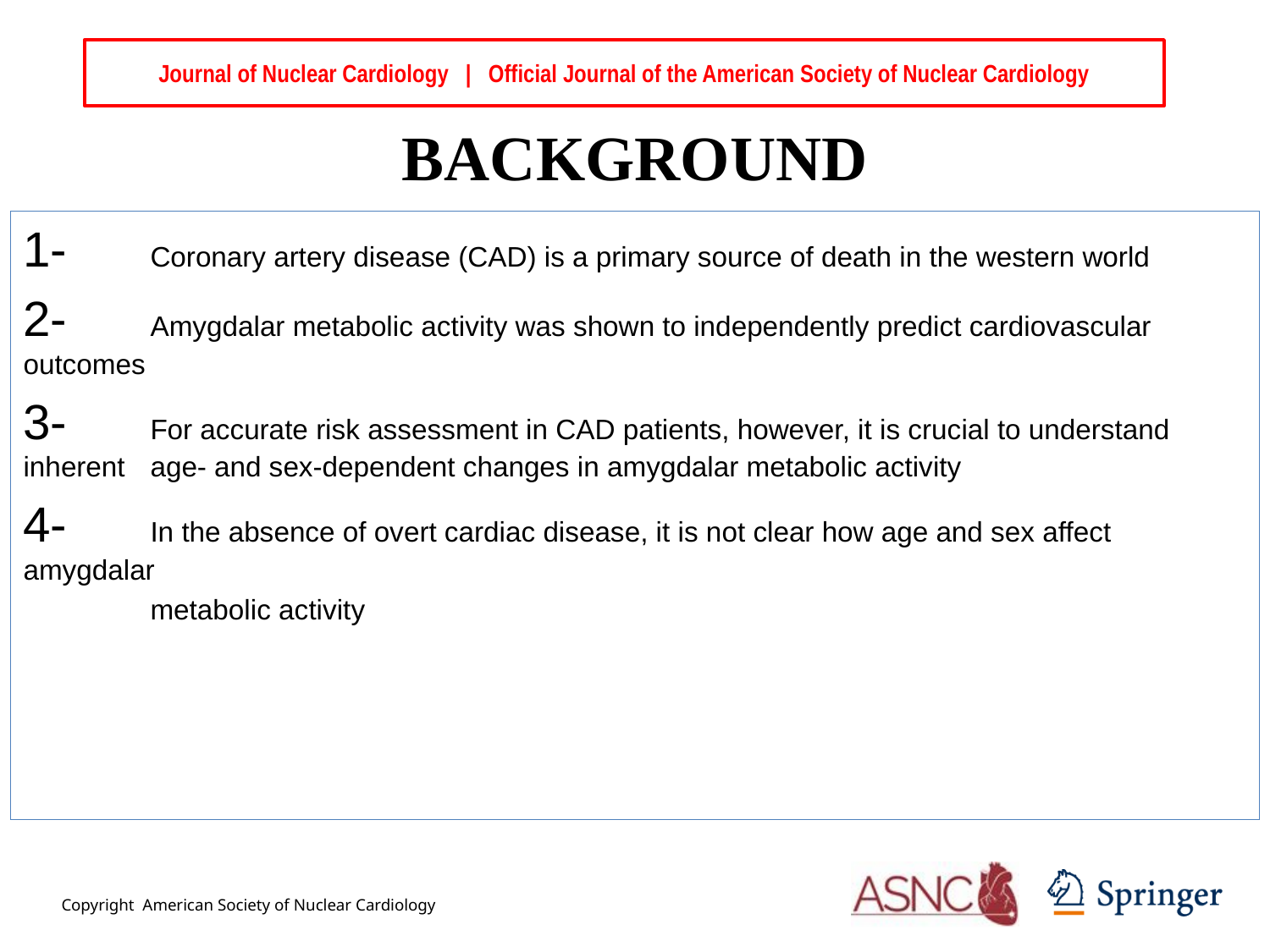

Journal of Nuclear Cardiology | Official Journal of the American Society of Nuclear Cardiology
# BACKGROUND
1- 	Coronary artery disease (CAD) is a primary source of death in the western world
2- 	Amygdalar metabolic activity was shown to independently predict cardiovascular outcomes
3- 	For accurate risk assessment in CAD patients, however, it is crucial to understand inherent 	age- and sex-dependent changes in amygdalar metabolic activity
4- 	In the absence of overt cardiac disease, it is not clear how age and sex affect amygdalar
 	metabolic activity
Copyright American Society of Nuclear Cardiology

## Slide 3
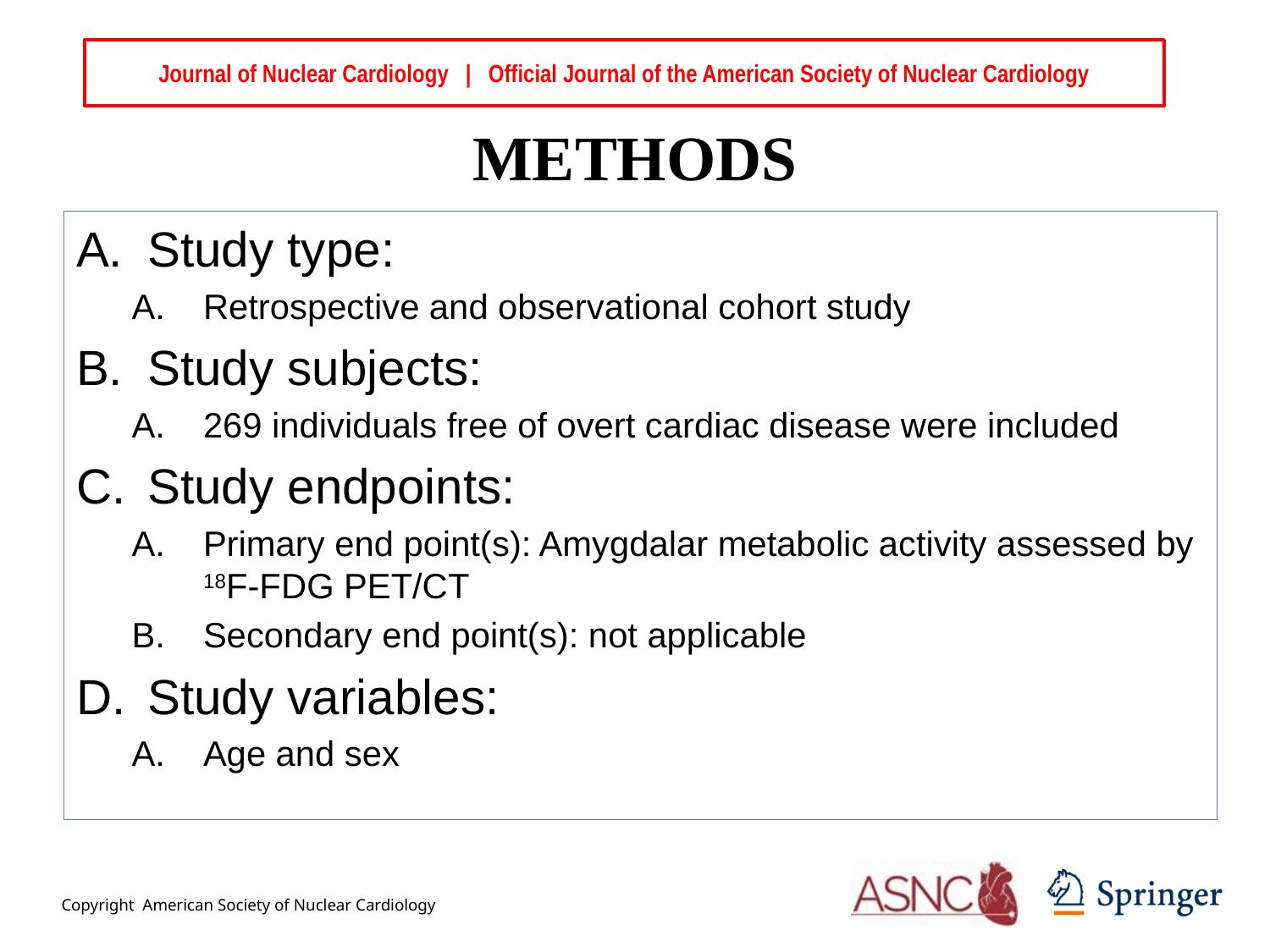

Journal of Nuclear Cardiology | Official Journal of the American Society of Nuclear Cardiology
# METHODS
Study type:
Retrospective and observational cohort study
Study subjects:
269 individuals free of overt cardiac disease were included
Study endpoints:
Primary end point(s): Amygdalar metabolic activity assessed by 18F-FDG PET/CT
Secondary end point(s): not applicable
Study variables:
Age and sex
Copyright American Society of Nuclear Cardiology

## Slide 4
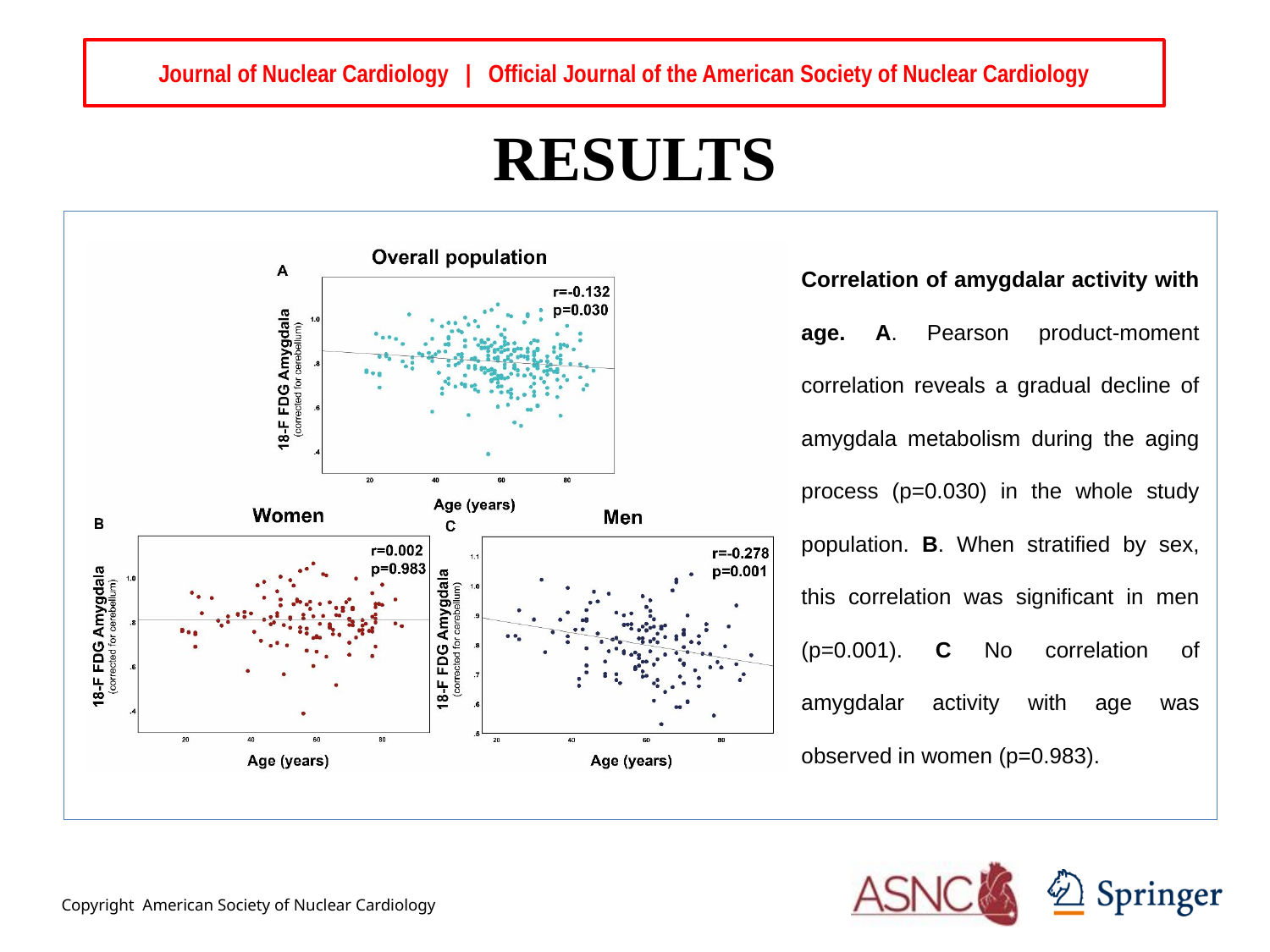

Journal of Nuclear Cardiology | Official Journal of the American Society of Nuclear Cardiology
# RESULTS
Insert a key table or a key figure
If figure, insert legend
Correlation of amygdalar activity with age. A. Pearson product-moment correlation reveals a gradual decline of amygdala metabolism during the aging process (p=0.030) in the whole study population. B. When stratified by sex, this correlation was significant in men (p=0.001). C No correlation of amygdalar activity with age was observed in women (p=0.983).
Copyright American Society of Nuclear Cardiology

## Slide 5
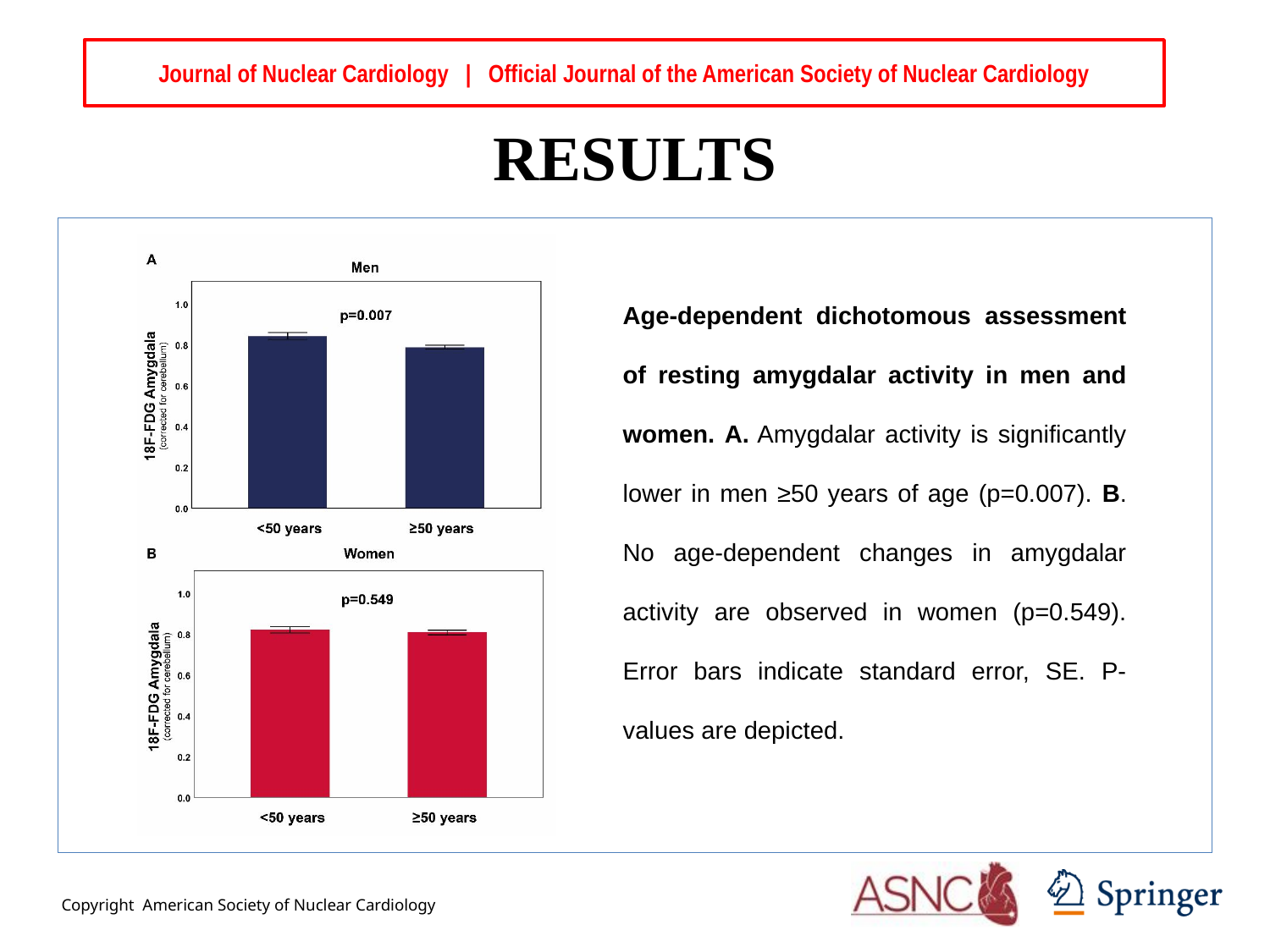

Journal of Nuclear Cardiology | Official Journal of the American Society of Nuclear Cardiology
# RESULTS
gggggggg
Age-dependent dichotomous assessment of resting amygdalar activity in men and women. A. Amygdalar activity is significantly lower in men ≥50 years of age (p=0.007). B. No age-dependent changes in amygdalar activity are observed in women (p=0.549). Error bars indicate standard error, SE. P-values are depicted.
Copyright American Society of Nuclear Cardiology

## Slide 6
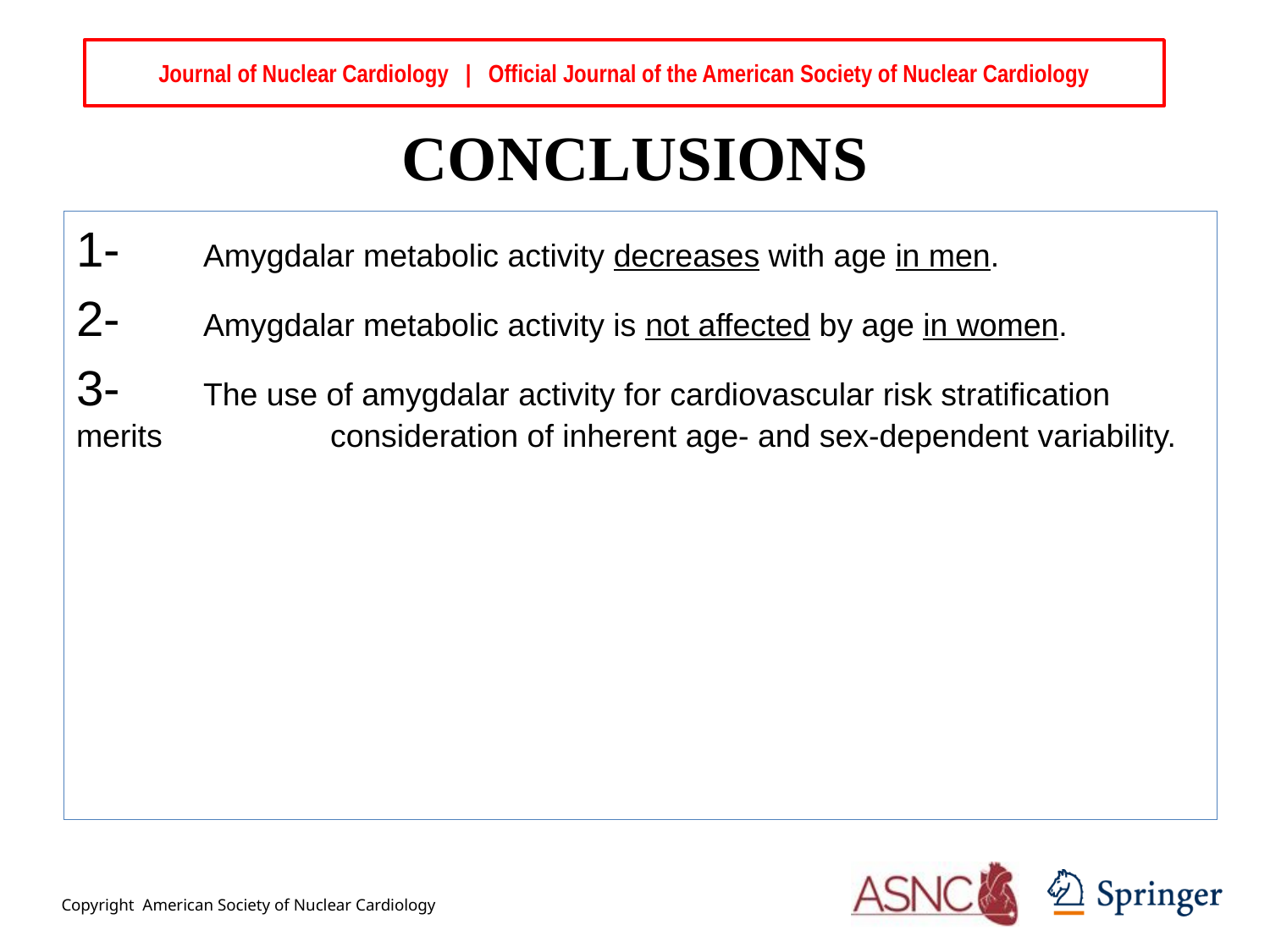

Journal of Nuclear Cardiology | Official Journal of the American Society of Nuclear Cardiology
# CONCLUSIONS
1- 	Amygdalar metabolic activity decreases with age in men.
2- 	Amygdalar metabolic activity is not affected by age in women.
3- 	The use of amygdalar activity for cardiovascular risk stratification merits 		consideration of inherent age- and sex-dependent variability.
Copyright American Society of Nuclear Cardiology
